# Correction: Use of the *Puccinia sorghi* haustorial transcriptome to identify and characterize AvrRp1-D recognized by the maize Rp1-D resistance protein

**DOI:** 10.1371/journal.ppat.1013022

**Published:** 2025-03-27

**Authors:** The PLOS Pathogens Staff

The affiliation for the 18^th^ author is incorrect. Doil Choi is not affiliated with #4 but with #5: Plant Immunity Research Center, Seoul National University, Seoul, Republic of Korea.

The publisher apologizes for the error.
