# Supplementary figures and images for: Use of the Puccinia sorghi haustorial transcriptome to identify and characterize AvrRp1-D recognized by the maize Rp1-D resistance protein
Source: PLoS Pathog. 2024 Nov 8;20(11):e1012662. doi: 10.1371/journal.ppat.1012662 (PMC11578463; doi:10.1371/journal.ppat.1012662)

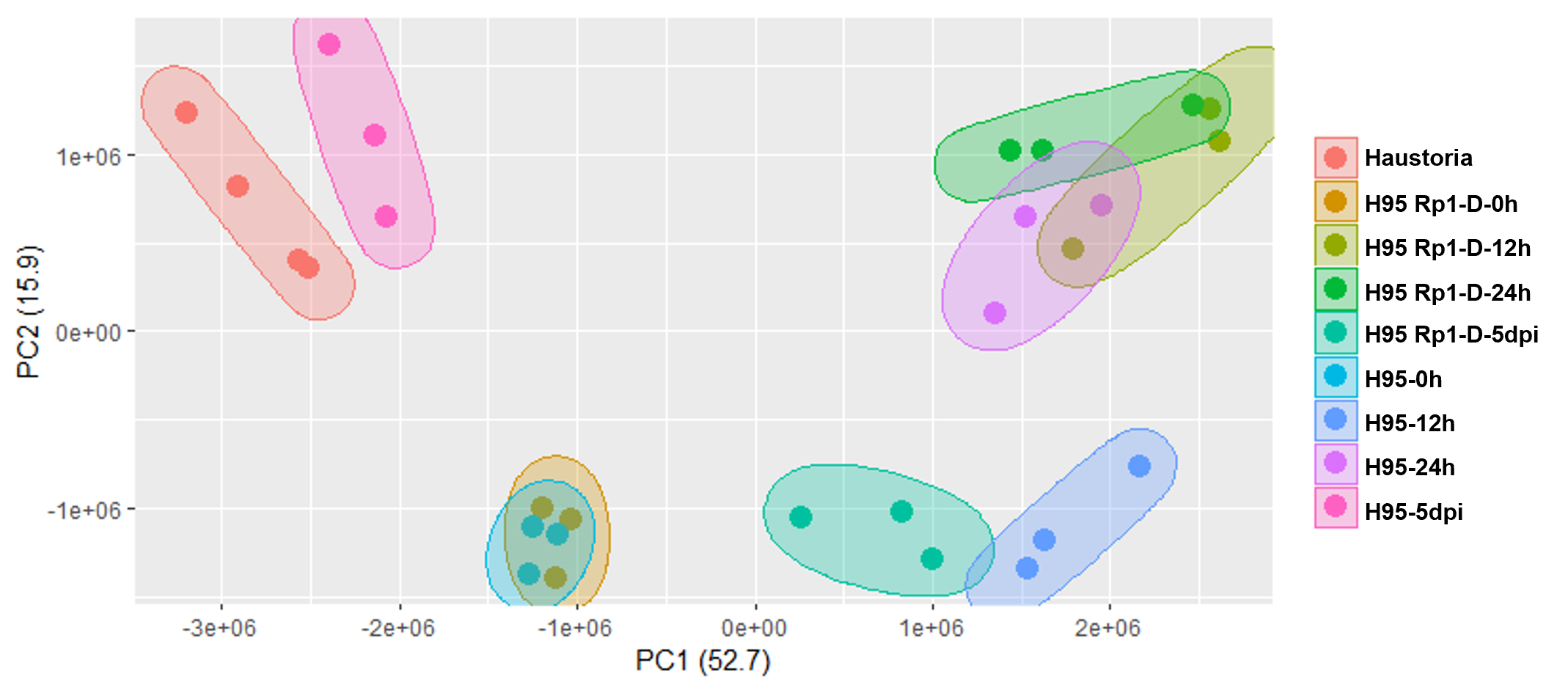

Supplement: S1 Fig — PCA plot of 72,538 P. sorghi transcripts expressed in H95, H95:Rp1-D, and in haustoria at different time points. (TIF) [file ppat.1012662.s001.tif]

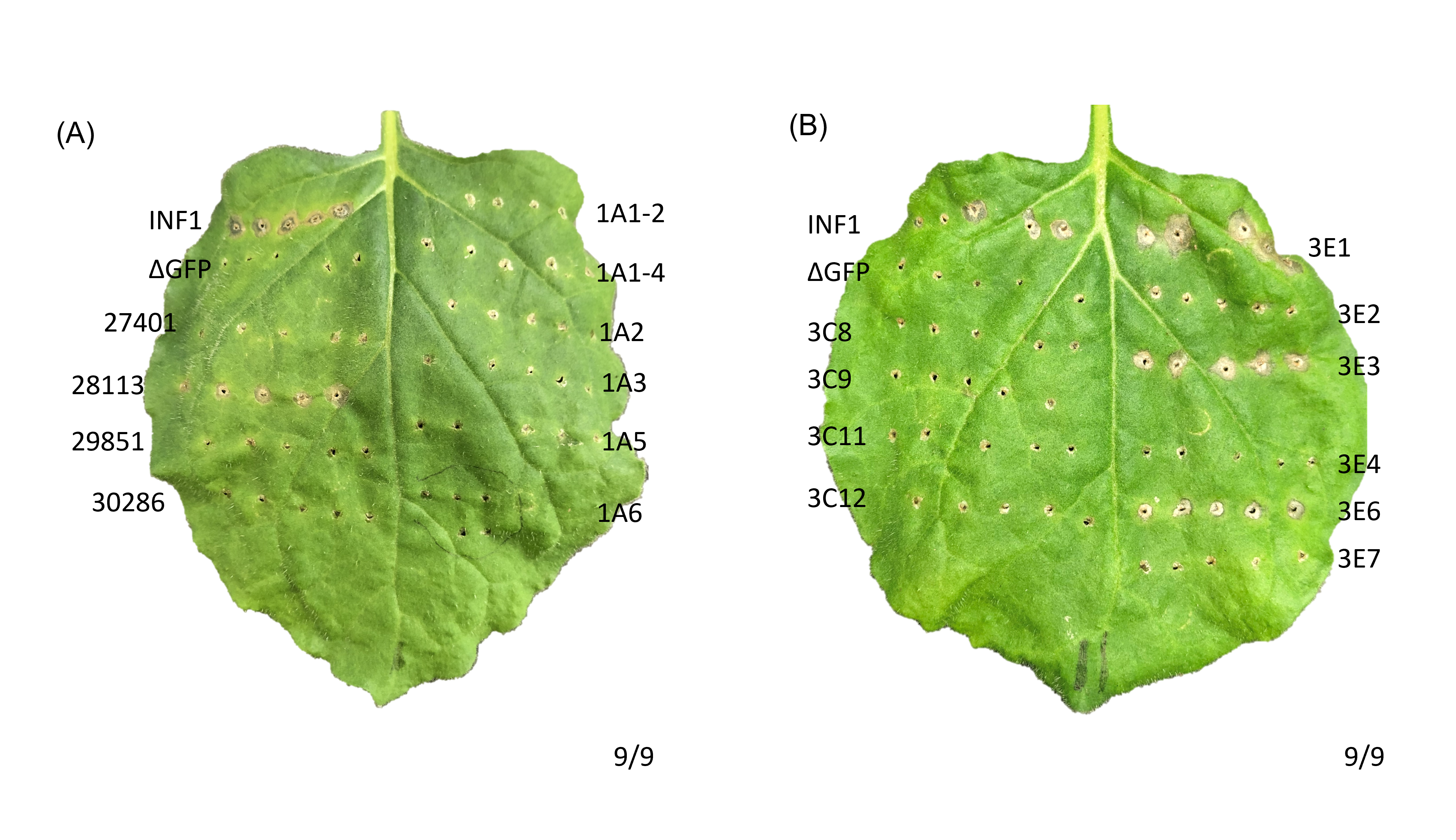

Supplement: S2 Fig — (A) and (B): Rp1-D was transiently expressed in the entire N. benthamiana leaf by agro-infiltration using a needle-less 1ml syringe and ten effector candidates expressed in a Potato Virus X-based pKW-LIC vector were then toothpicked into the leaf in a row of four 1 day afterward. pKW-INF1 was used as a positive control to induce cell death. pKW-ΔGFP was used as a negative control. A representative photo was taken at 5 dpi. The experiments were repeated three times with the same results. Three leaves for biological repeat were tested in each experiment. (TIF) [file ppat.1012662.s002.tif]

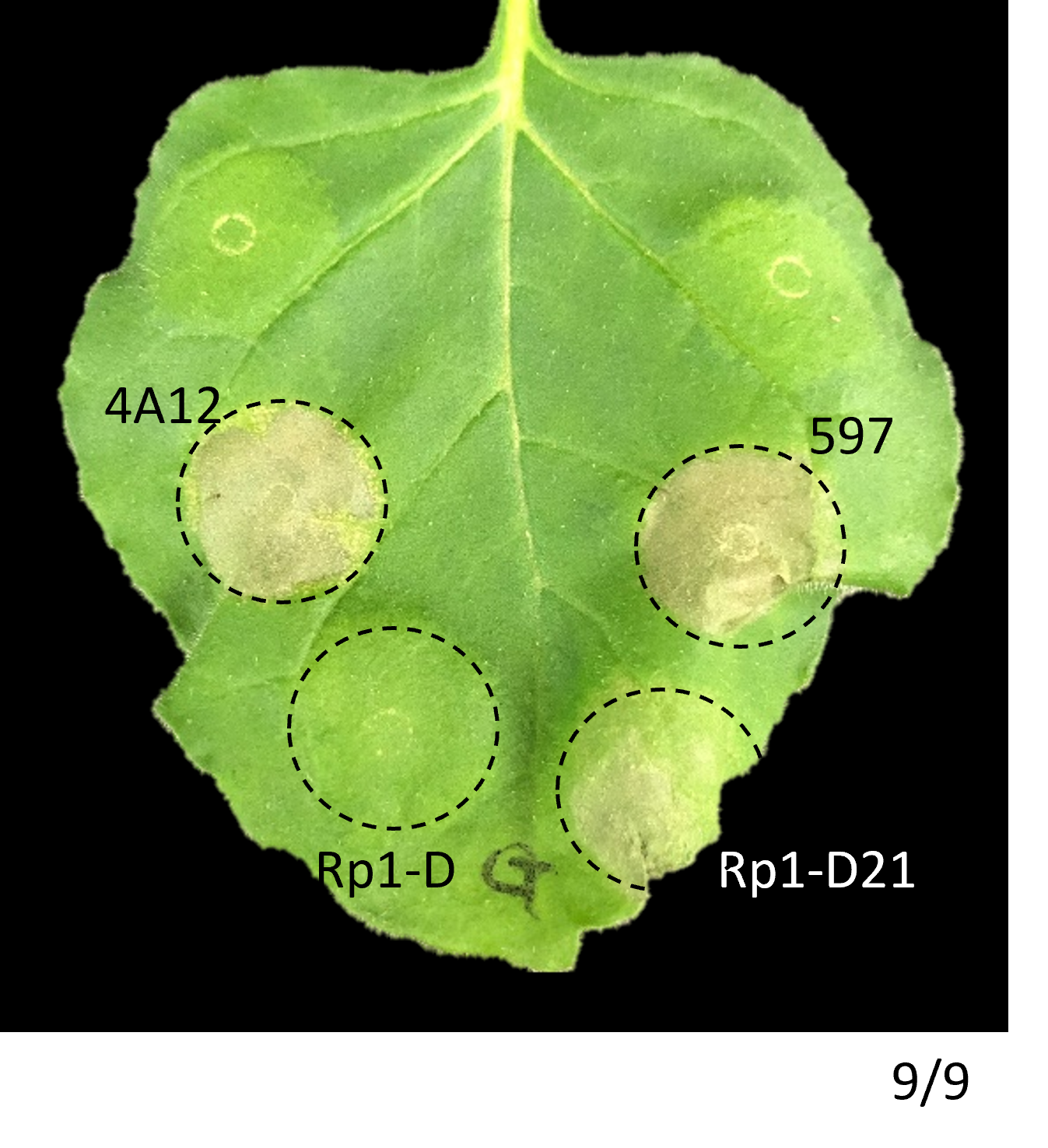

Supplement: S3 Fig — Rp1-D21:3xHA was used as a positive control. A representative photo was taken at 4 dpi. The dashed circles indicate areas of infiltration. 9 individual plants were infiltrated and showed similar results. (TIF) [file ppat.1012662.s003.tif]

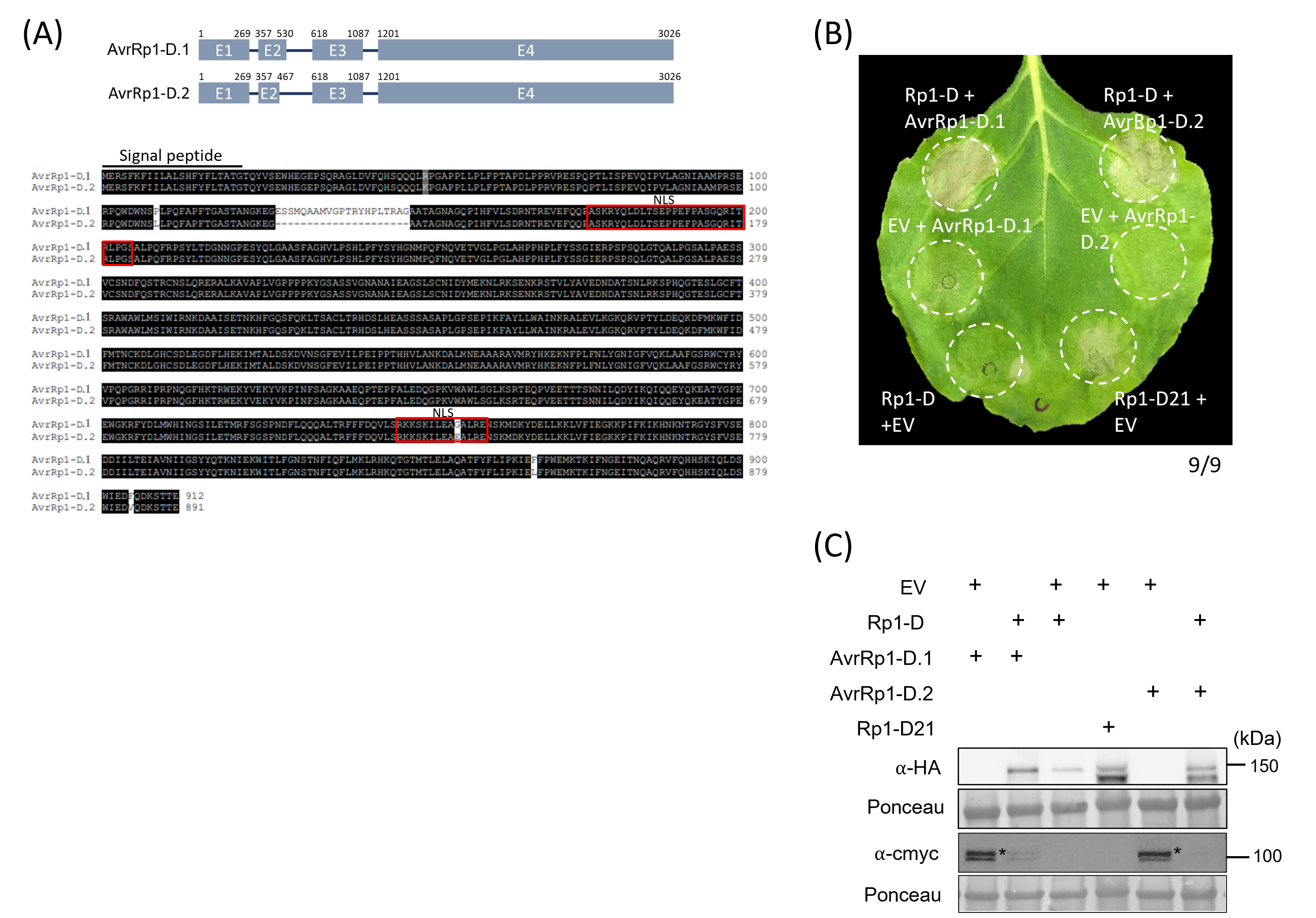

Supplement: S4 Fig — (A) An alignment of AvrRp1-D.1 and AvrRp1-D.2 amino acids. AvrRp1-D.2 has a 21 amino acid deletion in the ORF. (B) Co-expression of Rp1-D (fused with a C-terminal tag 3xHA) with AvrRp1-D.1 or AvrRp1-D.2 (fused with a C-terminal 4xcMYC). Representative leaf was photographed at 5 dpi. 9 individual plants were infiltrated and showed similar results. (C) Protein expression of Rp1-D and AvrRp1-D.1 variants transiently expressed in N. benthamiana. Total protein was extracted from agro-infiltrated leaves at 36 hpi, and anti-HA or anti-cMYC antibody was used to detect the expression of the fused proteins. The sizes of the proteins are indicated on the right. Ponceau S staining of the Rubisco subunit showed equal loading of protein samples. Three independent biological replicates were tested, and they showed similar results. (TIF) [file ppat.1012662.s004.tif]

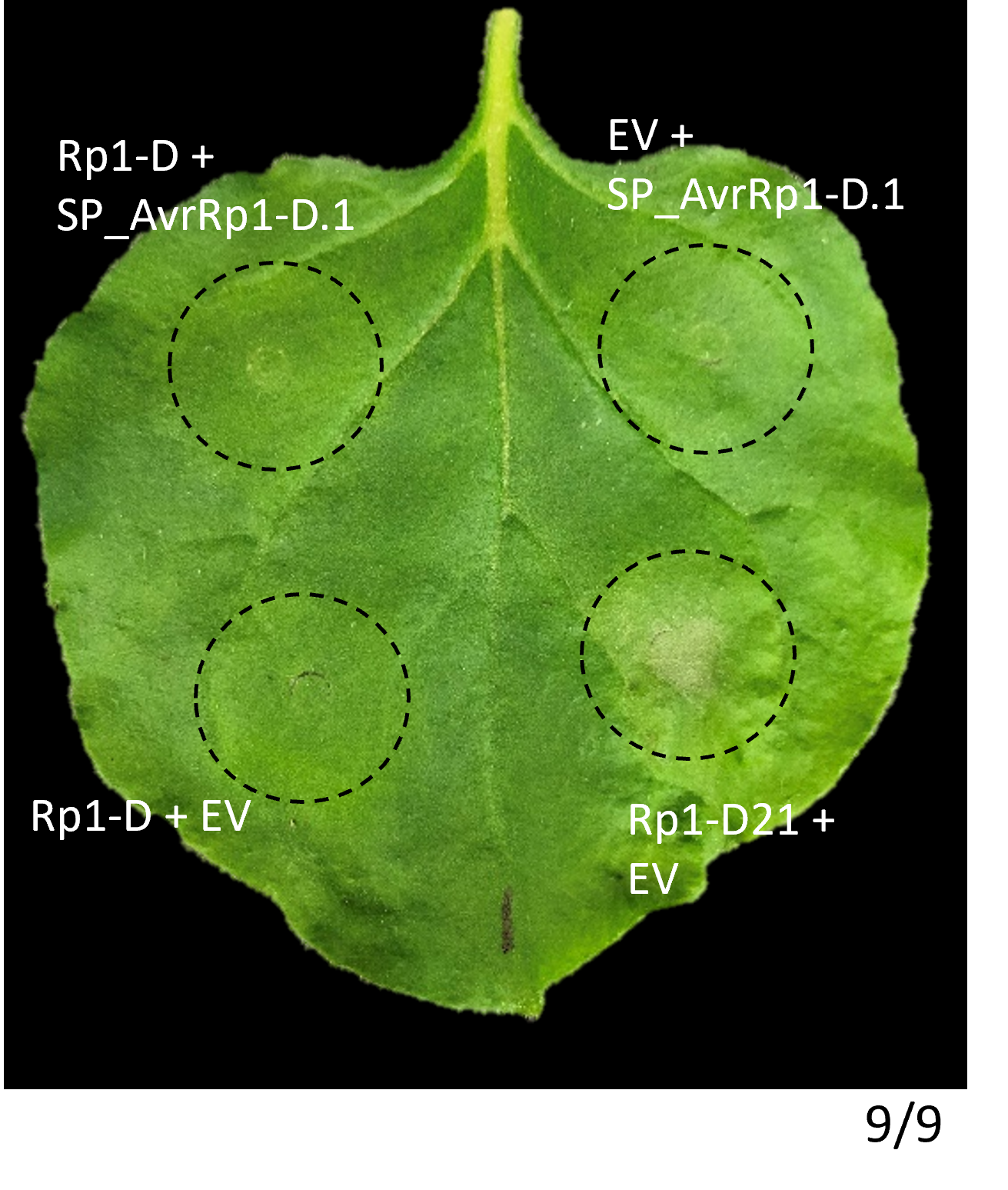

Supplement: S5 Fig — Co-expression of Rp1-D with AvrRp1-D.1 with signal peptide does not induce observable cell death in N. benthamiana. 9 individual plants were infiltrated and showed similar results. Empty vector (EV) was used as a negative control. Rp1-D21 expression was used as the positive control. (TIF) [file ppat.1012662.s005.tif]

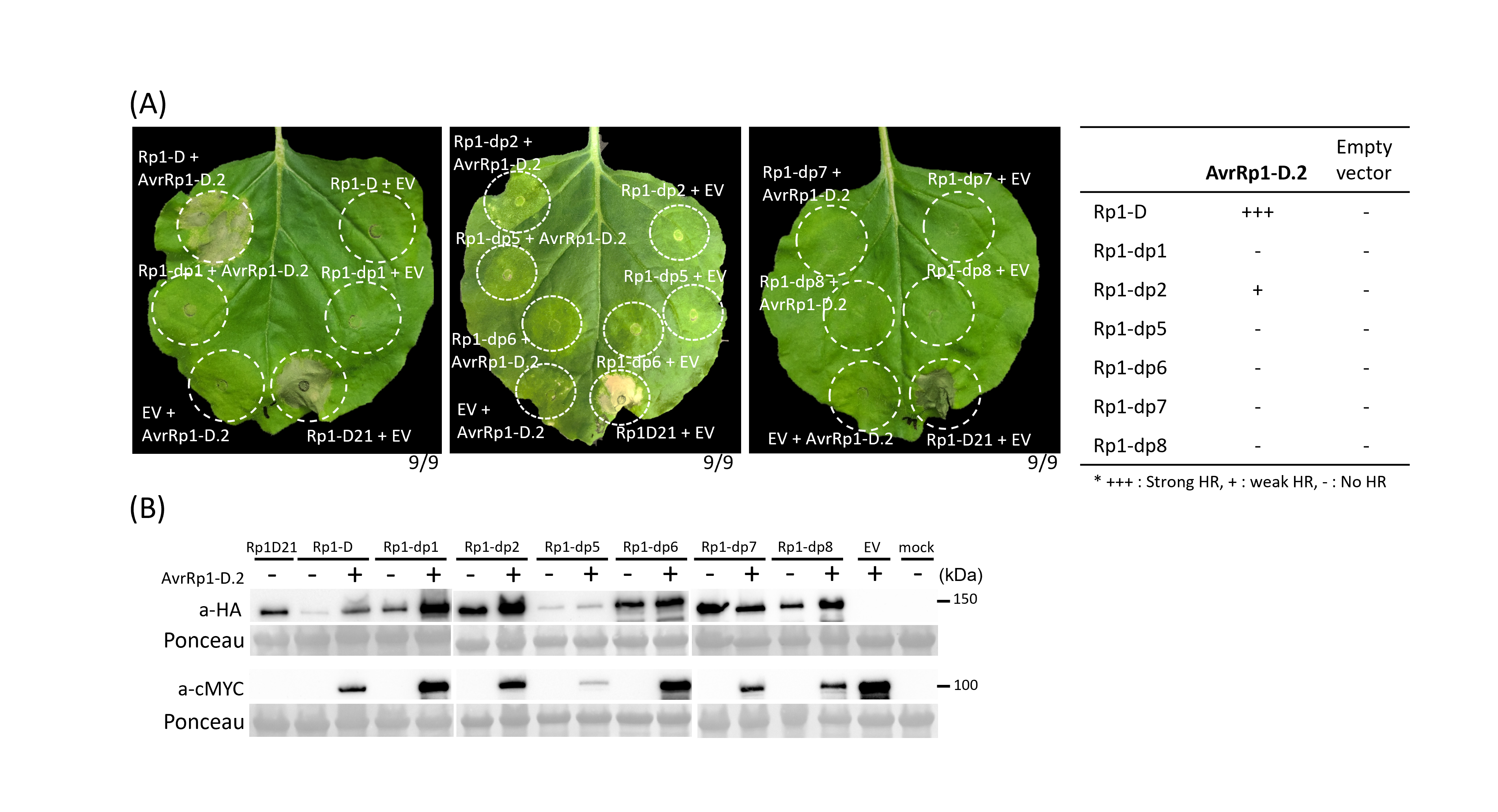

Supplement: S6 Fig — (A) Rp1-dp1, -dp2, -dp5, -dp6, -dp7, and -dp8 fused with a C-terminal tag 3xHA were co-infiltrated with AvrRp1-D.2 fused with a C-terminal 4xcMYC tag or EV in N. benthamiana. Representative photos were taken at 4 dpi. 9 individual plants were infiltrated and showed similar results. (B) Protein expression of Rp1 alleles and AvrRp1-D.2. Total protein was extracted from agro-infiltrated leaves at 36 hpi, and anti-HA or anti-cMYC antibody was used to detect the expression of the fused proteins. The sizes of the proteins were labeled on the right. Ponceau S staining of the Rubisco subunit showed comparative levels of protein samples in each lane. (TIF) [file ppat.1012662.s006.tif]

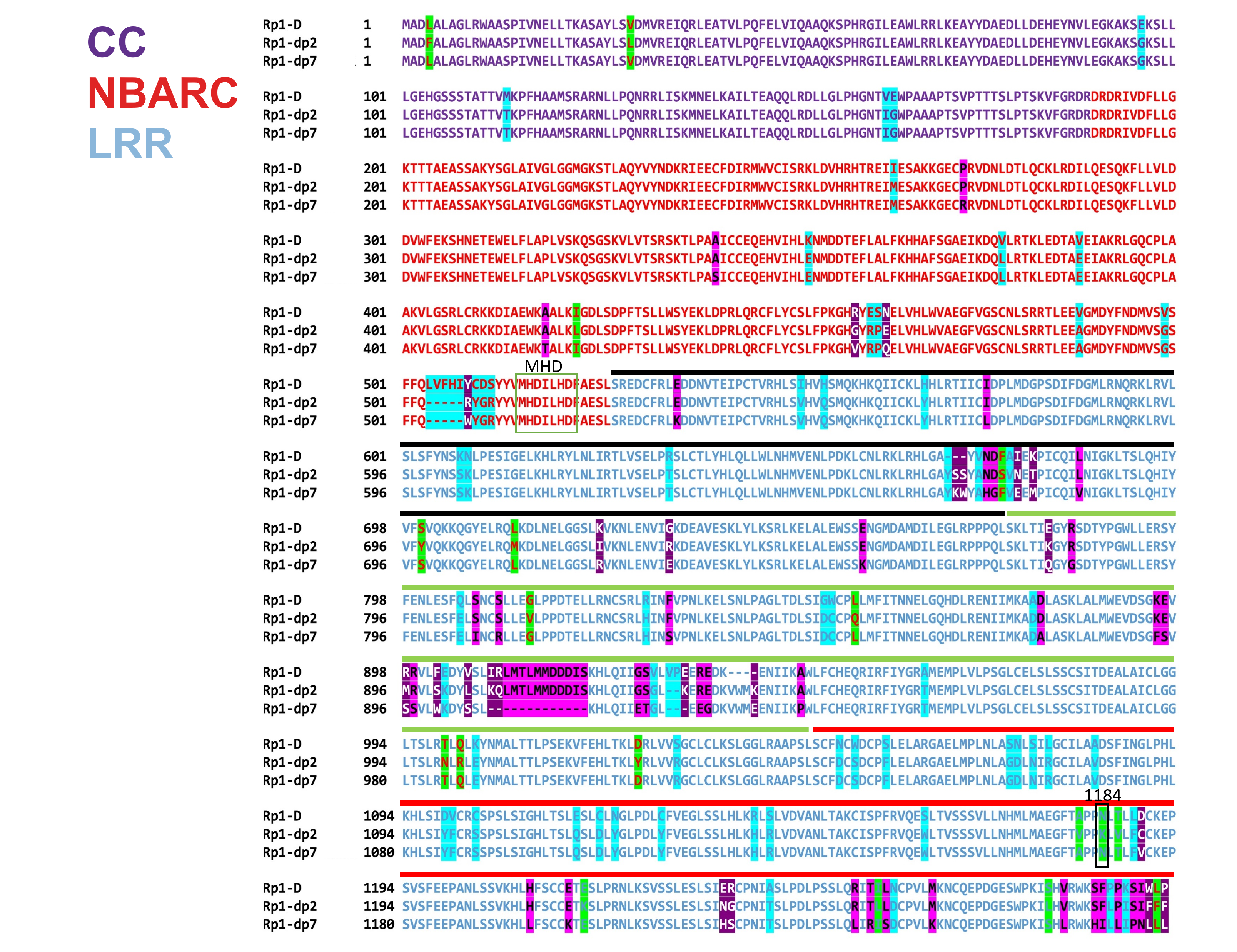

Supplement: S7 Fig — The CC, NB-ARC and LRR domains are indicated with purple, red and blue letters respectively. Black, green, and red lines indicate the LRR1, LRR2 and LRR3 regions shown. A green box indicates MHD and LHD motifs. All shades in different color indicate the difference among three alleles; Magenta: Rp1-D and -dp2 vs. -dp7, Green: Rp1-D and -dp7 vs. -dp2, Cyan: Rp1-D vs. -dp2 and -dp7, Purple: all three. (TIF) [file ppat.1012662.s007.tif]

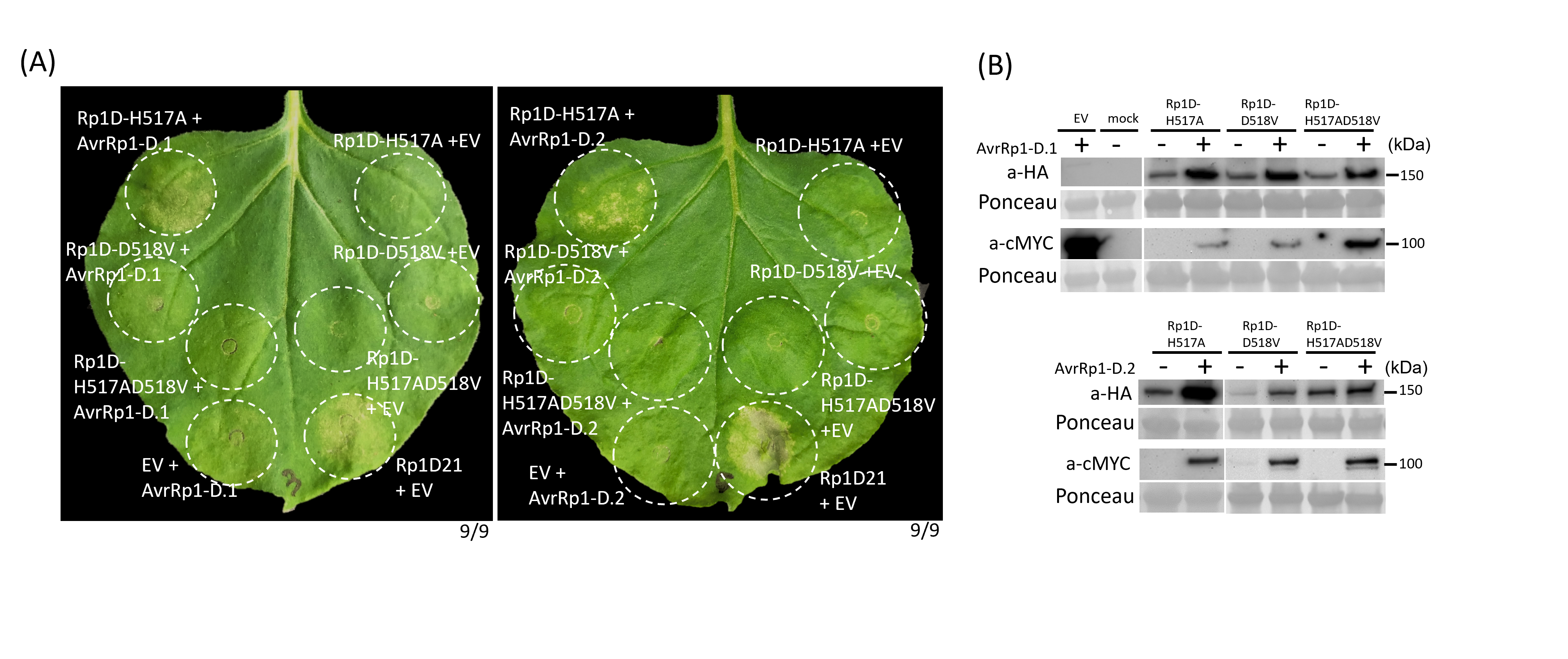

Supplement: S8 Fig — (A) All constructs tested were described previously (5). Rp1-D21 was used as a positive control. 9 individual plants were infiltrated and showed similar results. (B) The Rp1 allele mutants were fused with the C-terminal 3xHA and AvrRp1-D.1 and AvrRp1-D.2 were fused with the C-terminal 4xcMYC. Total protein was extracted from agro-infiltrated leaves at 36 hpi, and anti-HA or anti-cMYC antibody was used to detect the expression of the fused proteins. The sizes of the proteins were labeled on the right. Ponceau S staining of the Rubisco subunit showed equal loading of protein samples. (TIF) [file ppat.1012662.s008.tif]

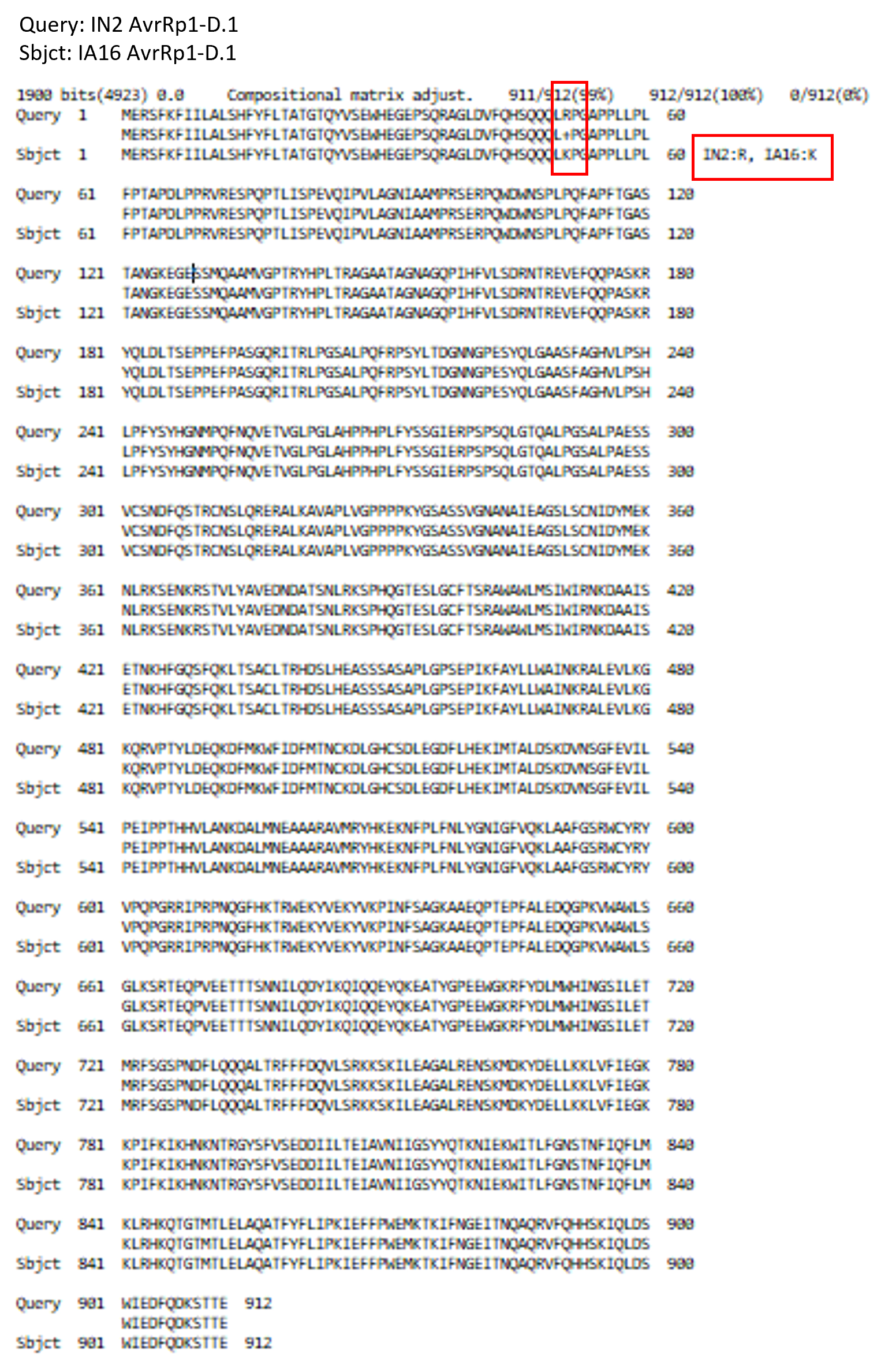

Supplement: S9 Fig — Only one amino acid is different in the alignment. Amino acid 51 is Arginine in IN2 and lysine in IA16. (TIF) [file ppat.1012662.s009.tif]

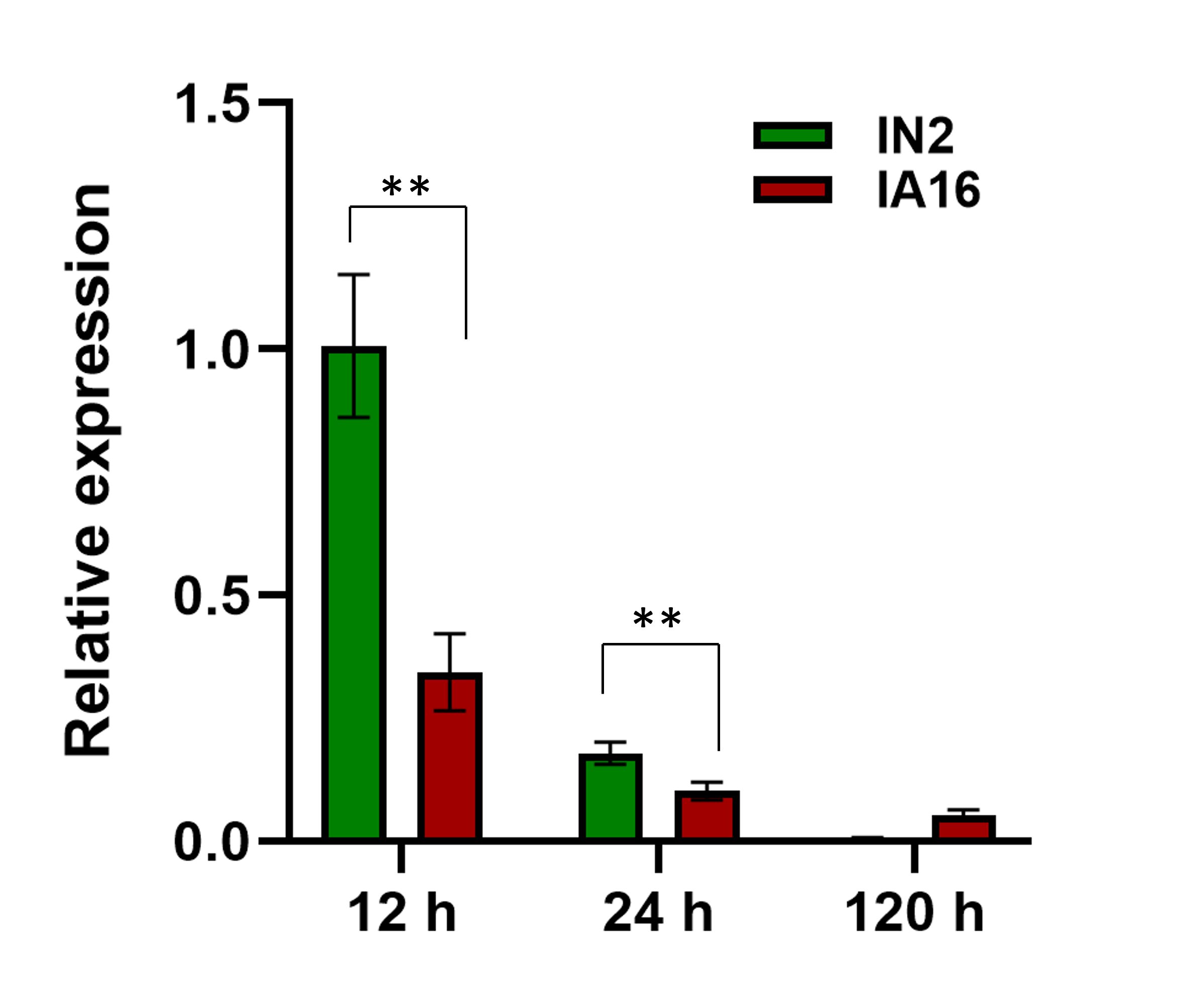

Supplement: S10 Fig — Expression level of AvrRp1-D.1 and AvrRp1-D.2 from the avirulent P. sorghi IN2 and the virulent P. sorghi IA16 (paired t-test, *p<0.05, **p<0.01) in the second repeat. One biological repeat was used for the graph. (TIF) [file ppat.1012662.s010.tif]
