# Supplementary material for: Use of the Puccinia sorghi haustorial transcriptome to identify and characterize AvrRp1-D recognized by the maize Rp1-D resistance protein
Source: PLoS Pathog. 2024 Nov 8;20(11):e1012662. doi: 10.1371/journal.ppat.1012662 (PMC11578463; doi:10.1371/journal.ppat.1012662)

Figure 2C

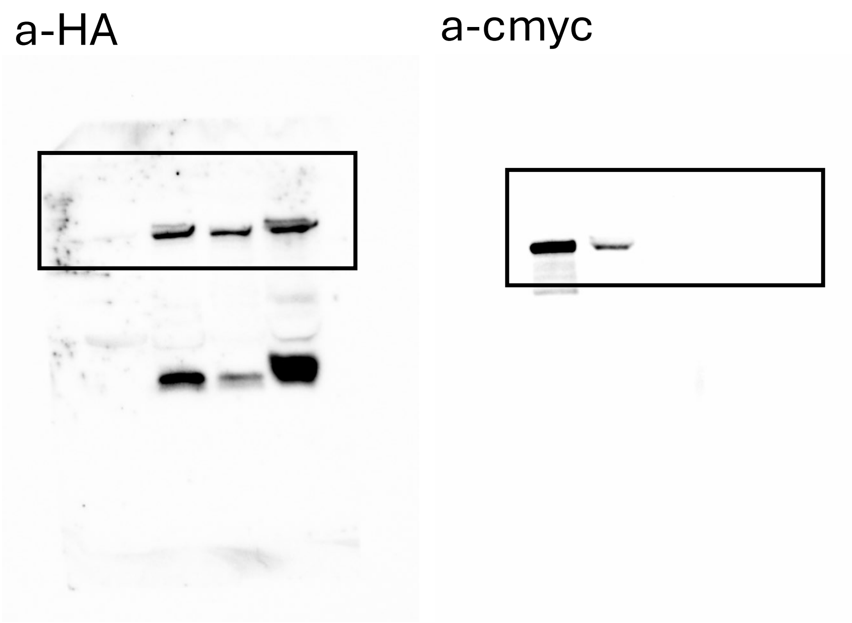

Figure 3C

a-HA

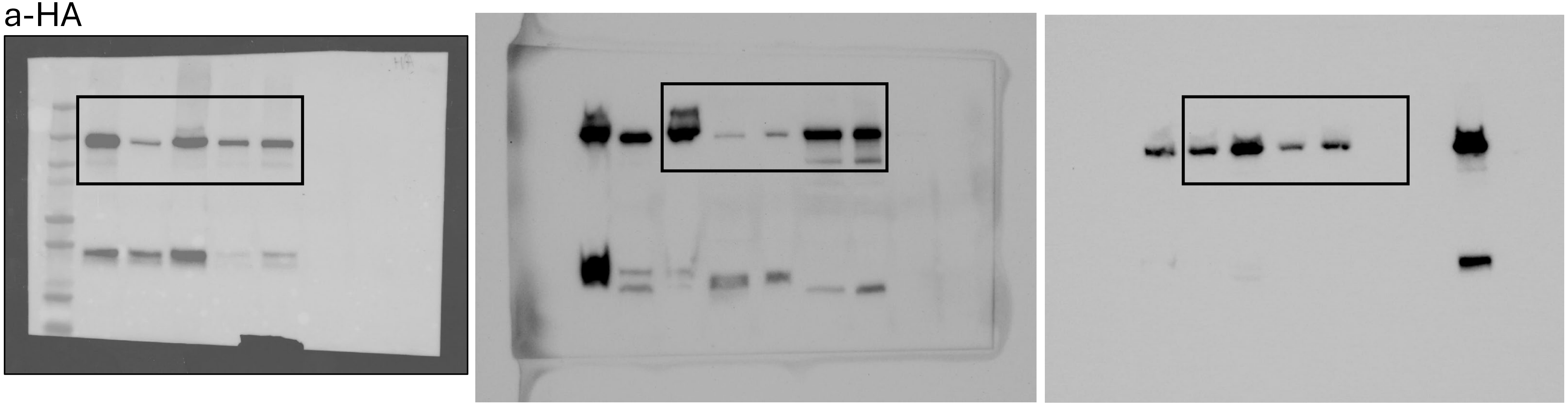

a-cmyc

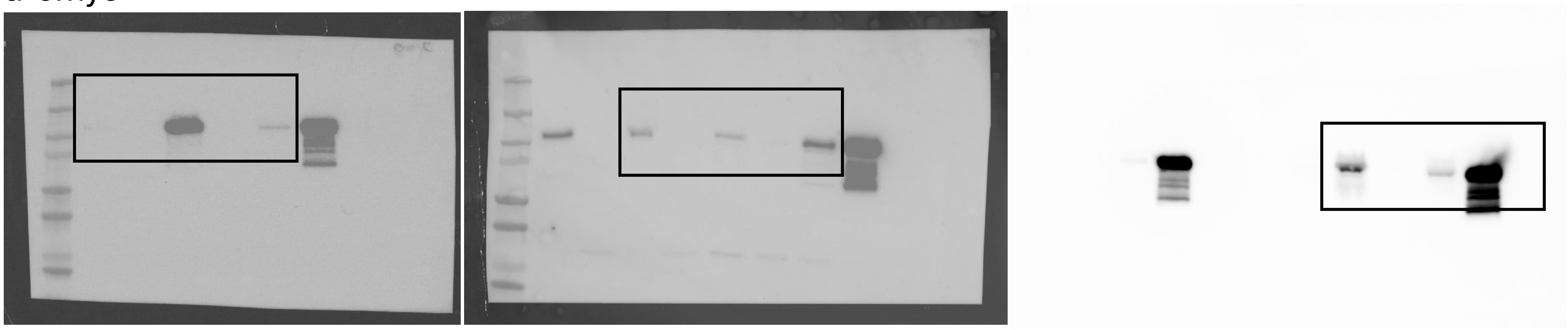

Figure 4B

a-HA

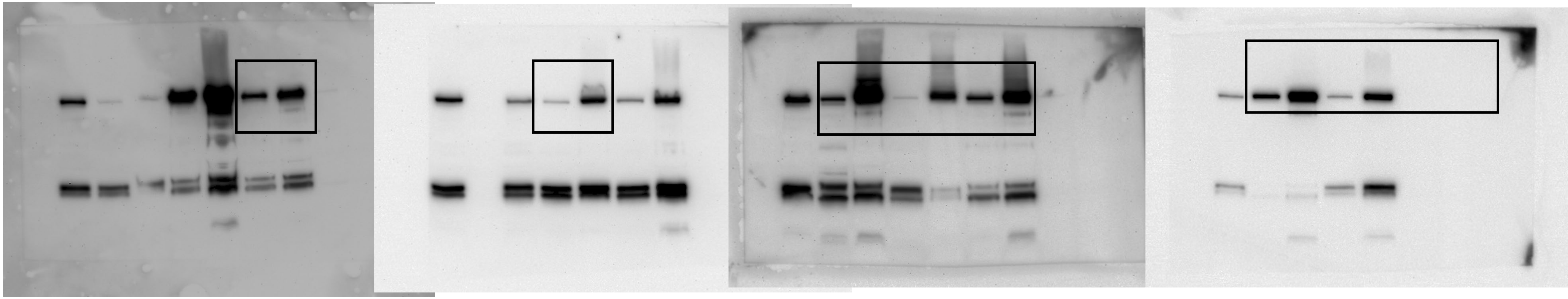

a-cmyc

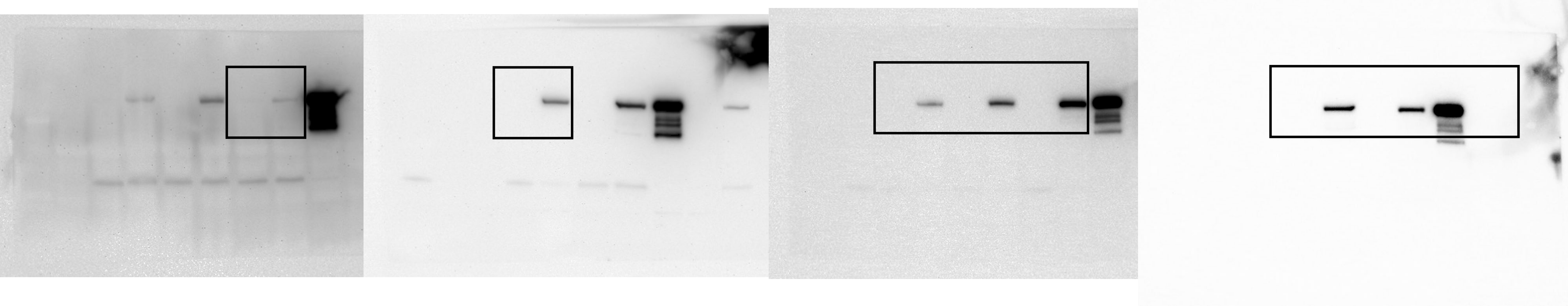

Figure 4C

a-HA

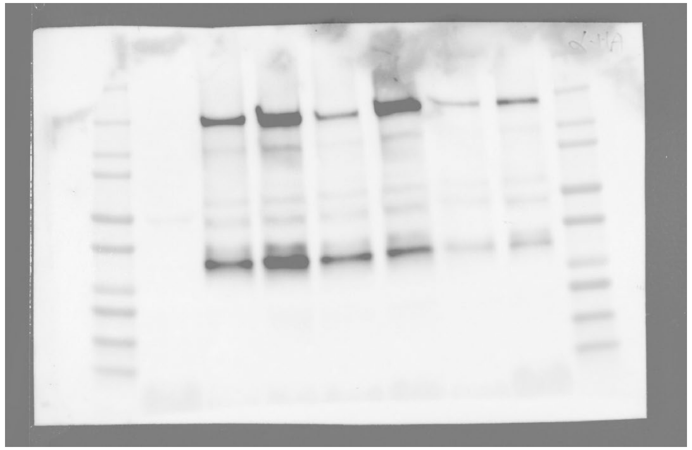

a-cmyc

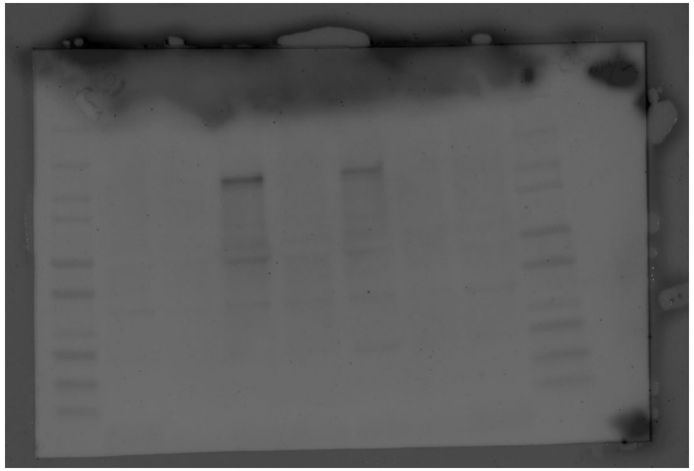

Figure 5C

a-HA

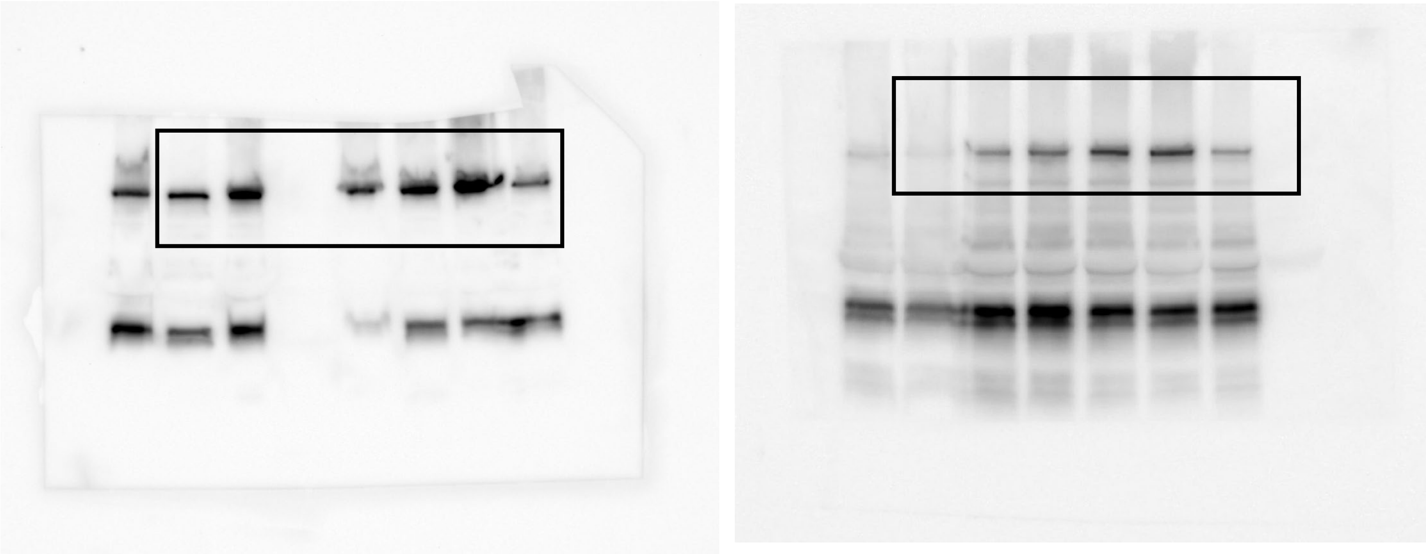

a-cmyc

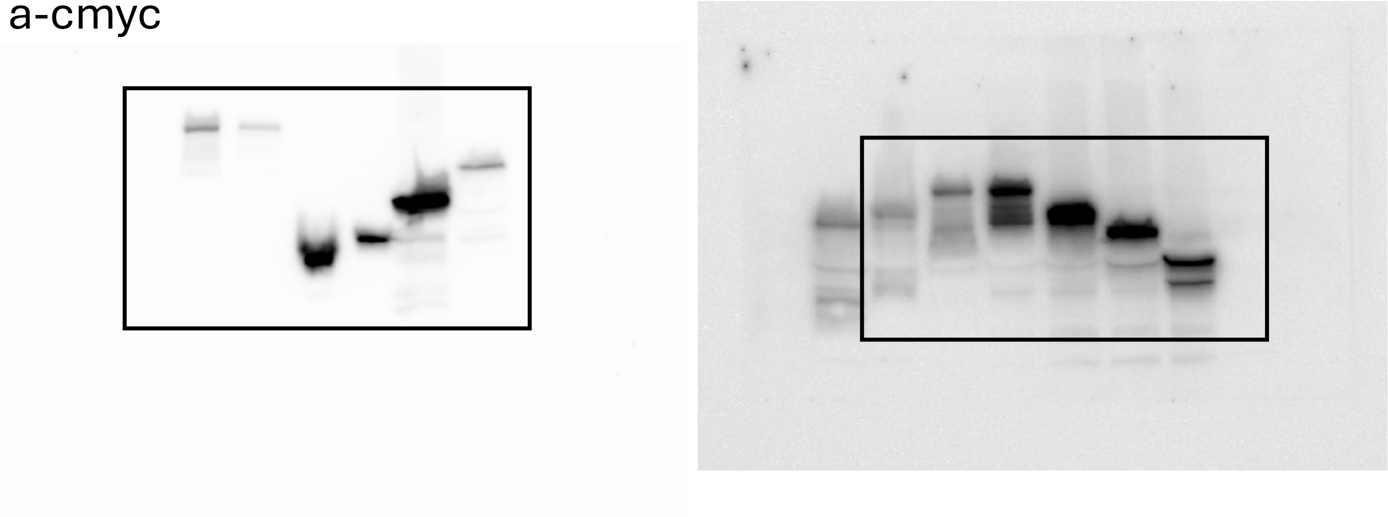

Figure 6C

a-HA

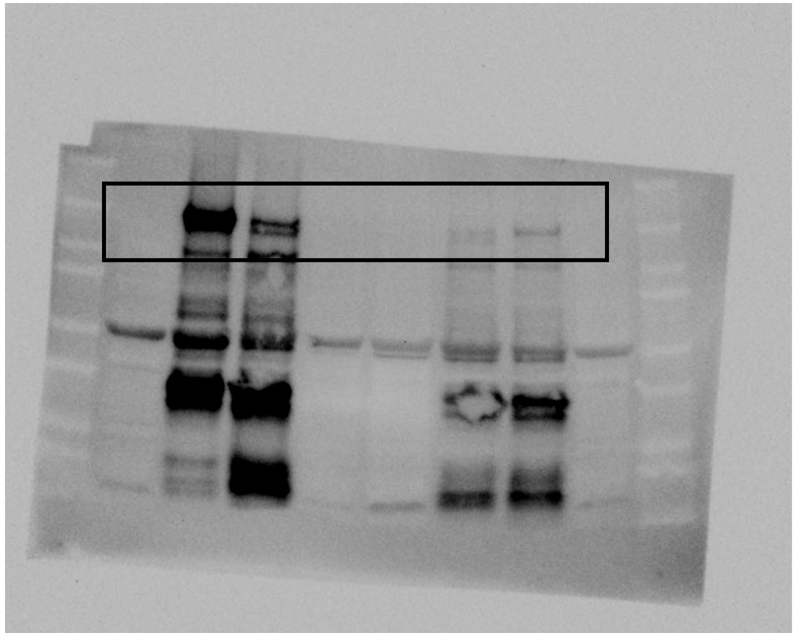

a-cmyc

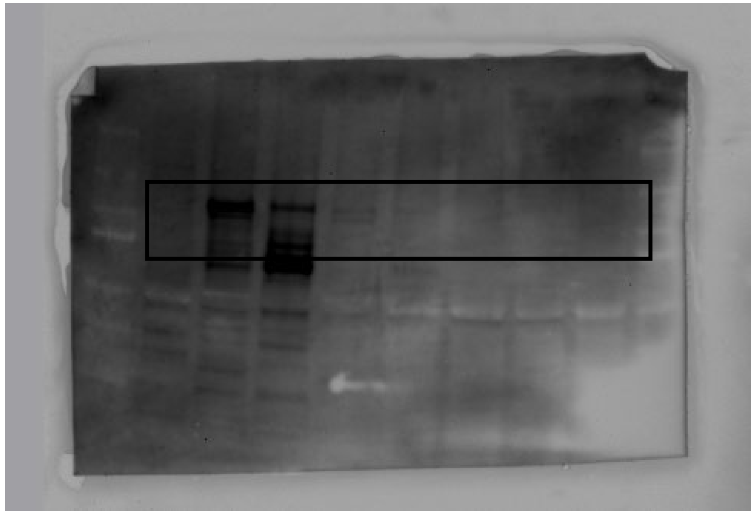

Supplementary figure 4(C)

a-HA

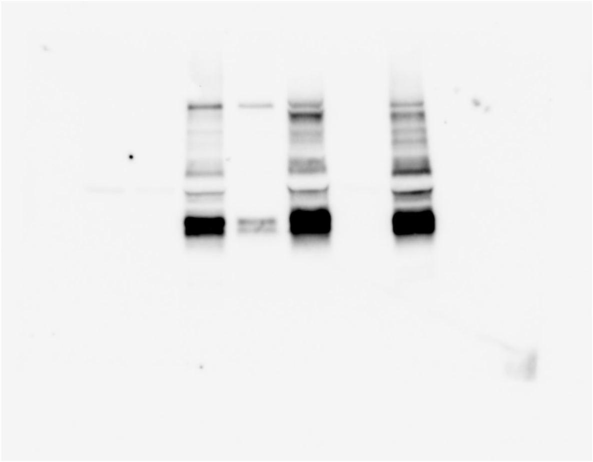

a-cmyc

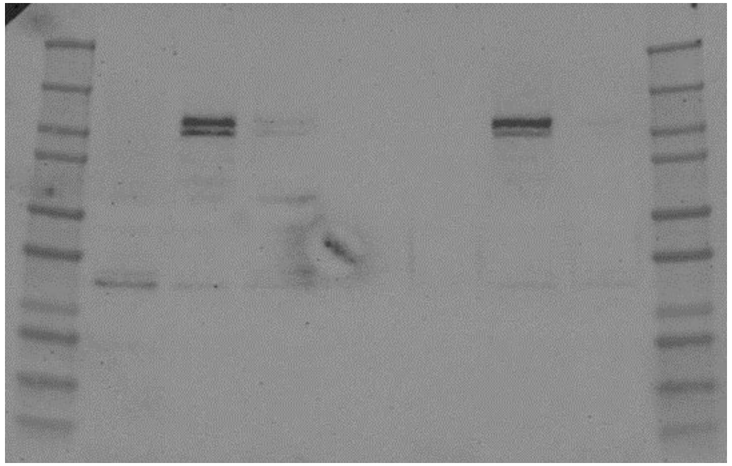

Supplementary figure 6B

a-HA

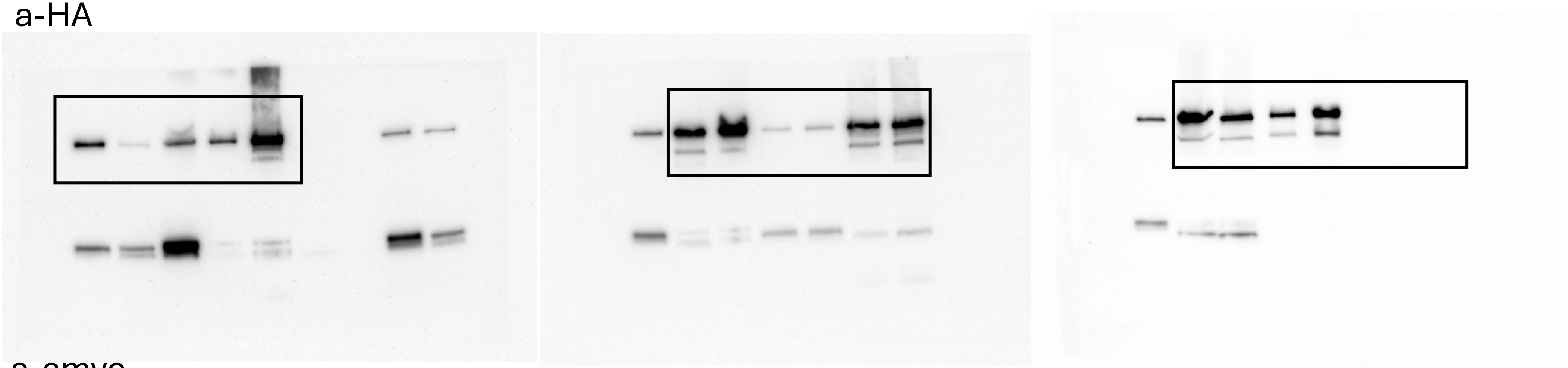

a-cmyc

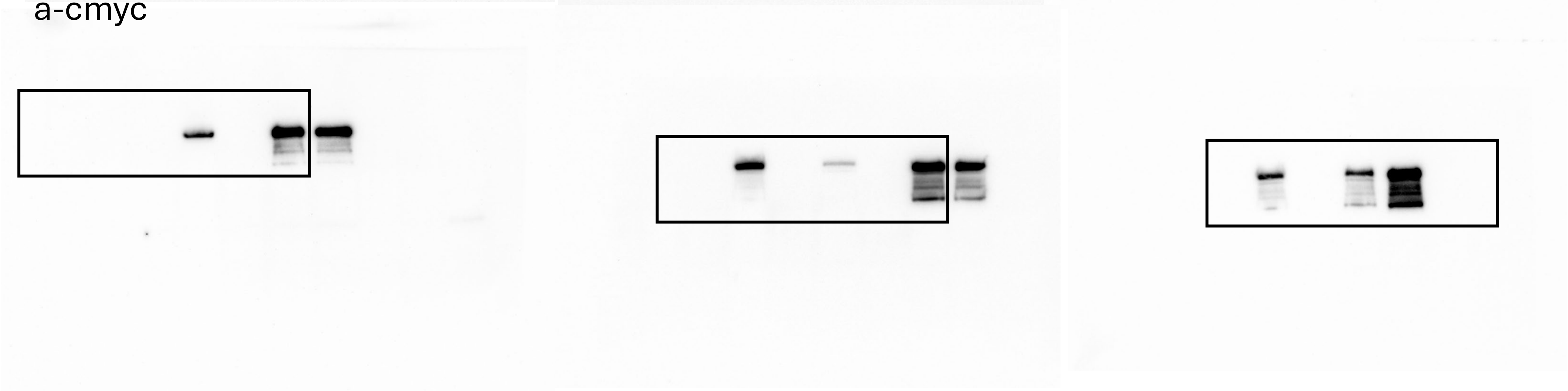

Supplementary figure 8B

a-HA

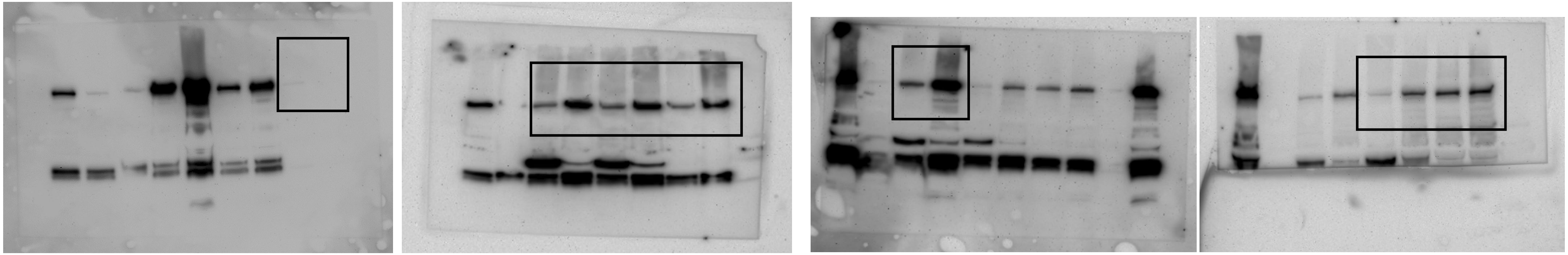

a-cmyc

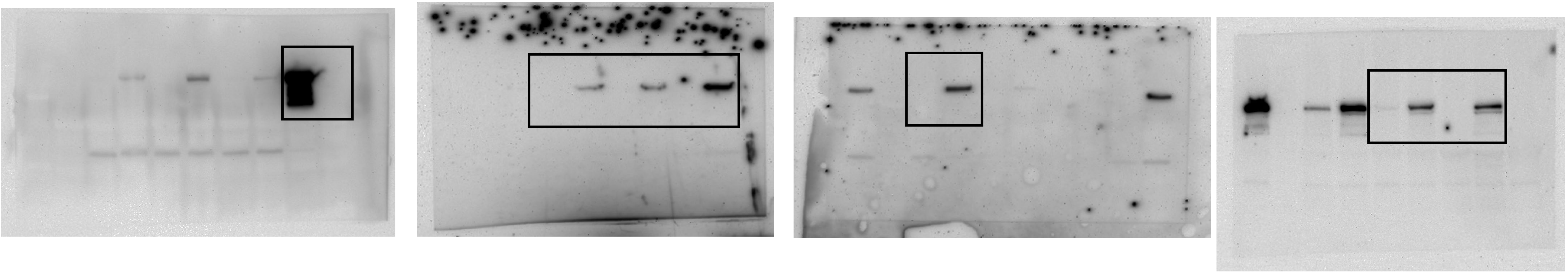

Supplement: S2 Data — (PDF) [file ppat.1012662.s018.pdf]
