# Supplementary material for: Use of the Puccinia sorghi haustorial transcriptome to identify and characterize AvrRp1-D recognized by the maize Rp1-D resistance protein
Source: PLoS Pathog. 2024 Nov 8;20(11):e1012662. doi: 10.1371/journal.ppat.1012662 (PMC11578463; doi:10.1371/journal.ppat.1012662)
Supplement: Attachment — Submitted filename: reviewer response_PLOS Pathogens_09022024.docx [file ppat.1012662.s019.docx]

Dear. Dr. Thomma,

We really appreciate the time you and the reviewers spent on this manuscript and are delighted that you felt it was appropriate for publication in PLOS Pathogens. Below, we have addressed the reviewers’ comments. In response to their comments, we have made some significant changes to the manuscript. We have added an additional supplemental figure and an additional element to the main figure.

Sincerely,

Saet-Byul Kim

-----------------------------------------------------------------------------------------------------

REVIEWER COMMENTS:

Reviewer #1: Yeast-to-hybrid assay: The authors should provide western blots of the fusion proteins used in the Y2H assay to show that they are indeed produced in yeast. Otherwise, absence of interaction cannot be concluded from the data provided in the manuscript.

A: We thank the reviewer for the suggestion. We have added western blots in Figure 4.

Lines 201-206: I have some reservations regarding the interpretation of the results in Figure 4(A). While the V17 construct, containing only the very C-terminal LRR repeats of Rp1-dp2, loses the ability to induce a hypersensitive response (HR), it's noteworthy that when a larger portion of the LRR repeat from Rp1-dp2 (V16) is introduced into Rp1-D, this chimeric construct displays a stronger HR reaction compared to the original Rp1-dp2. This observation suggests that not only the C-terminal part but also the additional portions of the LRR repeat are vital for the HR response. It implies that the presence of the C-terminal LRR in Rp1-D may not be strictly necessary for HR. Therefore, I suggest clarifying the role of the Cterminal LRR repeats in a set of swap experiments that complement the existing dataset with a swapping partner that, in contrast to Rp1-dp2, does not induce any HR response upon AvrRp1-D recognition (such as the Rp1-d7 allele also used in the Y2H assays).

A: We appreciate the reviewer’s point and agree that the additional regions of the LRR are critical for HR. To clarify this point, we generated three chimera mutants, V1_dp7, V16_dp7, and V17_dp7, containing a particular portion of Rp1-dp7. We have added supplementary figure 4, which shows the co-expression data of three chimera constructs with AvrRp1-D. As the V17 construct, containing the very C-terminal LRR of Rp1-dp2, does not induce HR, the V16_dp7 and V17_dp7 construct lose the ability to induce HR. In addition, V1_dp7 also does not induce HR upon AvrRp1-D recognition. As the reviewer mentioned, these results suggest that the C-terminal of LRR of Rp1-D is not solely responsible for inducing HR. We addressed this conclusion in Lines 246-253.

Reviewer #2: I have two suggestions for the authors to consider:
1. The authors described a modified protocol on haustoria isolation, which enables them to generate a haustorial transcriptome assembly and a list of predicted P. sorghi effectors. Presumably, this is a significant technical improvement for effector discovery. Since the authors have also generated RNA-seq data from infected tissues at different time points, it’d be very beneficial to run a parallel effector prediction with the RNA-seq data sets and compare the predicted effector lists from haustoria and infected tissues. It’d be interesting to understand the extent of improvement (e.g., how many additional effectors were predicted from the haustorial transcriptome assembly?).

A: We thank the reviewer for the suggestion. We compared the predicted effector lists from haustoria and infected tissues at three time points, as shown here. Only 1% of the RNA-seq data was used for de-novo assembly of effector sequences at 12 and 24 hours, while over 30% of the data was used at 120 hours. While 72,538 effector genes were predicted from haustoria, fewer effector genes were predicted in infected tissues—with 22,747, 21,932, and 49,433 genes at 12, 24, and 120 hours post-infection (hpi), respectively.

Interestingly, more than half of the predicted effector genes were uniquely expressed in each tissue type. Venn diagrams were created using sequences with a 100% match to compare the overlap of genes between tissues. However, we suspect that part of the reason that there were so many ostensibly unique genes was that errors occurred during the assembly process when sequences matched less than 100%, leading to their classification as different genes.

2. The authors identified an AvrRp1-D allele from a virulent isolate IA16, and the AvrRp1-DIA16 unexpectedly induced Rp1-D-dependent HR in N. benthamiana. The observation seems in contrary to the conclusion that this is the authentic Avr for Rp1-D. The authors speculated several hypotheses to explain the conflicts, but the most plausible explanation is expression variations between the two Avr alleles in infected tissues, which should be easy to compare by qRT-PCR or RNA-seq. I hope the authors can generate such expression data. Also, while Rp1-D + AvrRp1-D induces strong HR in N. benthamiana (comparable to Rp1-D21), Rp1-D + AvrRp1-D only gives a weak HR in maize protoplasts (much weaker than Rp1-D21, Fig. 2D). This raises the possibility that HR by Rp1-D/AvrRp1-D may not be strong enough to fully explain the resistance from Rp21 in maize (HR is “over-estimated” in N. benthamiana). The author may compare HR by Rp1-D/AvrRp1-D and Rp1-D/ AvrRp1-DIA16 in maize protoplasts. If Rp1-D/ AvrRp1-DIA16 gives a much weaker HR than Rp1-D/AvrRp1-D in maize, it’d support the authors’ main conclusion (AvrRp1-D is the Avr for Rp1-D). Otherwise, the authors should consider not to make such a strong conclusion (e.g., may call it a putative AvrRp1-D).

A: We appreciate the reviewer’s suggestions and concerns. We agree with the reviewer’s hypothesis of expression variations between avirulent and virulent alleles. To address this question, we performed qRT-PCR. We attempted to detect the only expression level of AvrRp1-D.1 based on the sequence unique in the N-terminus of AvrRp1-D.1. However, we found that the primer set designed for the specific detection of AvrRp1-D.1 was not sufficiently specific. Thus, we used a primer set that detects both AvrRp1-D.1 and AvrRp1-D.2 for the qRT-PCR analysis.

As hypothesized by the reviewer, the expression levels of *AvrRp1-D.1* and *AvrRp1-D.2* from the avirulent isolate IN2 were approximately three-fold higher than those of AvrRp1-DIA16 from the virulent isolate IA16 (Fig. 6B and S10 Fig). This result suggests that higher expression of AvrRp1-D.1 and AvrRp1-D.2 genes may contribute to avirulence during the infection of Rp1-D plants. We addressed this result in lines 296-300. Although we presented one dataset for the graph, similar results were obtained in the second repeat. Upon analyzing the Ct values from the qRT-PCR, we observed that the expression of the *P. sorghi* succinate dehydrogenase gene, used as an endogenous reference gene, showed significantly different values between the two sets at the same time point. We assumed that the endogenous reference gene expression of two isolates in infected tissues of susceptible plants should be similar at the same time point. So this indicates that the infection was not uniform across the leaf. Based on this observation, we hypothesize that the uneven leaf infection caused by the rubbing method may be responsible for this difference. We couldn’t combine two datasets for the graph generated by normalizing the expression of the same endogenous reference gene.

We agree with the reviewer’s points about the protoplast assay result.

However, the probability of Rp1-D and AvrRp1-D being transfected into one protoplast, resulting in HR, might be lower than the case where a single Rp1-D21 gene results in HR. Due to the lab issue, we were not able to perform the protoplast assay to compare HR by Rp1-D/AvrRp1-D and Rp1-D/ AvrRp1-DIA16 in maize protoplasts. We performed the protoplast assays; the first author has moved from NC State University to a new position at the University of Nebraska. Since the protoplast assay is technically challenging, it has not been possible yet to set up this system at her new location. On the protoplast assay, we disagree with the reviewer that the strength of the response in the assay can give us an idea of how effective the HR is in a natural context, which is a very different situation. While we have not performed the protoplast assay with the IA16 allele for the reasons explained, we would argue that if, as we suggest, the virulence of strain IA16 is due to low expression of Avr-Rp1D rather than differences in the encoded Avr proteins, then this experiment would not give us the answer is desired.

**Part III – Minor Issues: Editorial and Data Presentation Modifications**
Please use this section for editorial suggestions as well as relatively minor modifications of existing data that would enhance clarity.

Reviewer #1:

Figure 1 panel D, what does relative expression level mean? To what are the read counts normalize? Please specifiy.

A: We thank the reviewer for pointing out our mistake. We have deleted ‘relative’ in the figure legend 1. We used DESeq2 to take as input a matrix of “read counts” mapped to genes. We used a Python script (prepDE.py) that can be used to extract this read count information directly from the files generated by StringTie. The input data was non-normalized, as suggested by DESeq2. To normalize for sequencing depth and RNA composition, DESeq2 uses the median of ratios method.

Figure 4, panel A: AvrRp1-D with construct Rp1-D21 is indicated as +++ but there is no HR visible on the picture

A: We thank the reviewer for pointing out our mistake. We used the wrong picture and changed it to the new one.

Figure 2~~1~~, panel D, was the empty vector only transfected once? Because the individual datapoints indicated values for biological replicates are not shown.

A: We appreciate the reviewer’s point. The empty vector was transfected three times independently. We have updated the graph with the individual data points.

Methods Line 386-398, Please provide more detail in the description of the in-silico predictions utilized in this study. Specify the thresholds applied in the different prediction tools such as SignalP and EffectorP etc. . Additionally, provide details how this information was utilized to predict and prioritize the candidate effector list for experimentation.

A: We thank the reviewer for the suggestion and agree that this section requires more detail. We have added the thresholds in the following section. However, TMHMM v2.0, ApopalstP, TargetP-2.0, EffectorP v2.0, and EffectorP v3.0 do not provide the default thresholds on their websites or papers, so we could not provide that information in the manuscript.

Line 429: “SignalP4.1 identified the signal peptide (SP) with the default cutoff of 0.45 (57). SignalP 4.1 was first applied to 72,538 transcript contigs to identify effector candidates carrying signal peptides. TMHMM v2.0 was subsequently utilized to sort out the effector proteins without transmembrane domains. The transmembrane domains were identified by TMHMM v2.0 with the default threshold (58). As a result, we identified 1,761 effector candidates that have signal peptides but do not have a transmembrane domain. The other tools for assisting the annotation included were ApoplastP (59), TargetP-2.0 (60), EffectorP v2.0 (61), and EffectorP v3.0 with the default cutoff (62). BlastP (63) was used to detect maize homologs using the best hit in the 1e-5 value (Zm-B73-REFERENCE-NAM-5.0_Zm00001eb.1).”

Furthermore, provide details regarding the threshold employed to identify homologs against maize and specify the source of the reference maize genome utilized.

A: We have added the following to specify the threshold.

Line 436: “using the best-hit in the 1e-5 value.”

Methods Line 409: Please specify the primer sequences that you used to create the effector library using LIC cloning.

A: We appreciate the reviewer’s point. We have added the following.

Line 461: 251 effector candidates were amplified with primers that included 17 and 15-nucleotide tails: forward, 5’-CCAATCCCTCTACG-ATG-gene specific sequence-3’ and reverse, 5’-TATCCTCCTACG-stop codon-gene specific sequence-3’ for use in the ligation-independent cloning (LIC) method.

Method Line: 443, where the plasmids containing R and AVR or EV plasmides mixed in equimolar ratio? Please specify.

A: We have added the following.

Line 496: Six µL of the LUC reporter construct, 14 µL of the Rp1-D construct, and 10 µL of the EV or AvrRp1-D construct (LUC/NLR/Avr ration=3:7:5) were mixed for each transfection.

2) Transcirptome assembly: the transcriptome assembly should be provided in a readily accessible format, such as a multifasta file (machine readable format) on a repository (Zenodo for instance) instead of providing it as part of an excel file.

A: We appreciate the reviewer’s suggestion. We have added transcriptome assembly as a fasta format file in the supporting data 1.

Discussion:
Line 283-285, did you provide this information somewhere in the results and method section? Please specify what you mean by 94%? Where 94% of transcripts identical between the two methods?

A: We appreciate the reviewer's point. We have added the following in the result section.

Line 159: The sequence analysis indicated that of the 241 de novo assembled sequences, 202 showed approximately 94% or higher identity with the sequenced sequences.

Line 301-304, here it would be interesting if you discuss your own findings a bit more carefully. Your finding that the NLS signal is not required for Rp1-D activation supports a cytoplasmic NLR activation, or at least it does not contradict it.

A: We appreciate the reviewer’s point. To address whether the NLS of AvrRp1-D is required for Rp1-D-mediated HR, we have assessed the subcellular localization patterns of the deletion constructs of AvrRp1-D in N. benthamiana (Fig 5). We think the nuclear localization of AvrRp1-D might not be essential for causing cell death with Rp1-D since most deletion constructs of AvrRp1-D are localized in the nucleus. We have added the following.

Line 340: the nuclear localization of AvrRp1-D might not be essential to induce Rp1-D-mediated HR.

Line 325: “data not shown”, is not accepted anymore. If you have, the data why not show it for instance in a supplementary figure.

A: We thank the reviewer for pointing out our mistake. We performed the RT-qPCR to assess the expression level of AvrRp1-D alleles. We have added Fig 6 and supporting fig 10.

1) Throughout the manuscript: uncropped western blots should be provided as supplementary material.

A: We appreciate the reviewer’s suggestion. We have added all uncropped western blots in the supporting data 2.

Supporting Figure 4, panel C, loading control for one of the western blots is missing.

A: We appreciate the reviewer’s point. We have updated it to supporting fig 4.

Line 119, for a better understanding of the experimental workflow; please specify how the information of the Signalp4.1 and TmHmm prediction were used to filter the transcripts.

A: To help the reviewer understand, we updated the details in line 429.

Line 429: SignalP4.1 identified the signal peptide (SP) with the default cutoff of 0.45 (57). SignalP 4.1 was first applied to 72,538 transcript contigs to identify effector candidates carrying signal peptides. TMHMM v2.0 was subsequently utilized to sort out the effector proteins without transmembrane domains. The transmembrane domains were identified by TMHMM v2.0 with the default threshold (58). As a result, we identified 1,761 effector candidates that have signal peptides but do not have a transmembrane domain.

Reviewer #2: (No Response)
